# Social judgments on abortion and contraceptive use: a mixed methods study among secondary school teachers and student peer-counsellors in western Kenya

**DOI:** 10.1186/s12889-020-08578-9

**Published:** 2020-04-15

**Authors:** Miranda Håkansson, Stephanie Super, Monica Oguttu, Marlene Makenzius

**Affiliations:** 1 Department of Global Public Health, Karolinska Institutet, SE-171 77 Stockholm, Sweden; 2 Kisumu Medical Education Trust (KMET), Kisumu, Kenya

**Keywords:** Stigma, Abortion, Contraception, Adolescent pregnancy, Comprehensive sexuality education

## Abstract

**Background:**

In Kenya, unsafe abortion is the leading cause of maternal deaths in adolescent girls aged 15–19 years, and a majority did not use a modern contraceptive before becoming pregnant. The aim of this study was to explore attitudes related to abortion and contraceptive use among secondary school teachers and student peer-counsellors in a low-resource setting in western Kenya.

**Methods:**

A mixed methods design, combining a questionnaire-survey and focus group discussions (FGDs), was utilised to explore attitudes to abortion and contraceptive use among teachers (*n* = 15) and student peer-counsellors (*n* = 21) at a secondary school in Kisumu, Kenya. First, two Likert scale questionnaires were used: a modified version of the Stigmatising Attitudes, Beliefs and Actions (SABA) scale and the Contraceptive Use Stigma (CUS) scale. Secondly, four FGDs were conducted. Descriptive statistics and Abductive Thematic Network Analysis (ATNA) were used to analyse the data.

**Results:**

Overall, *Social judgments on abortion and contraceptive use* were found among teachers and student peer-counsellors, with similar patterns between sexes. *Christian and cultural values;* A majority, 28/36 considered abortion a sin, and chastity and purity before marriage were highly valued feminine ideals. *Discrimination and isolation;* 18/36 believed that a girl who has had an abortion might be a bad influence on other girls, and 13/35 stated that an adolescent girl cannot decide for herself if to use a contraceptive method. *Conflicting views on abortion and contraceptives;* A third (11/34) believed that contraceptives may cause infertility, and its use was related to promiscuity. Girls associated with abortion and contraceptive use were considered immoral, lacking parental guidance, and were used to represent bad examples in school. Although conflicting views were present, *sexuality was considered a taboo topic,* which left adolescents ignorant on contraceptive use.

**Conclusions:**

Adolescent girls associated with abortion and contraceptive use are at risk for social judgements and discrimination, by both peers and teachers. Sexual and reproductive health training needs to be implemented in teacher education to increase knowledge on adolescent sexuality, abortion and contraceptive use to improve adolescents’ sexual health and decrease the stigma.

**Trial registration:**

This was a prestudy nested in a cluster randomised intervention study, registered on February 28, 2017, at ClinicalTrials.gov (NCT03065842).

## Background

Around the world, unsafe abortion continues to be an important cause of maternal morbidity and mortality. Nearly 7 million women in low-income and middle-income countries were treated for complications of unsafe abortion in 2012 [1]. According to recent estimates, unsafe abortion may account for 15% of maternal deaths globally [2]. Contributing factors include restrictive abortion laws, limited access to safe sexual and reproductive health care, and in many cases a lack of knowledge about sexuality, reproduction and contraception [3, 4]. In Africa, adolescent girls (15–19 years) are the most affected population, accounting for 25% of unsafe abortions in the region [5]. Young pregnant girls have an increased risk of maternal morbidity and mortality compared to women of other ages [6, 7]. Furthermore, social consequences of teenage pregnancy, such as school dropout, result in lower educational attainment and decreased social opportunities [8].

Abortion stigma is widely acknowledged and has been defined as “a negative attribute ascribed to women who seek to terminate a pregnancy that marks them, internally or externally, as inferior to ideals of womanhood” [9]. Various labels such as promiscuous, sinful, selfish, irresponsible or murderous are applied to women who have an abortion. These negative stereotypes are sustained by fear of discrimination and social exclusion which keep women and others from speaking out in support of those who abort [9]. In some communities, laws and policies prevent provision of contraceptives to unmarried adolescents or to those under a certain age. However, even where there are no legal restrictions, misconceptions and stigmatising views among adolescents, parents and health care providers continue to block adolescents from using effective contraceptive methods [10, 11].

Kenya has a young population with an estimated 10.5 million adolescents aged 10–19 years, corresponding to 22.5% of the country’s total population [12]. The maternal mortality ratio has been declining during the last decade to reach 510 in 2015 [13]. Since 2010, a revised abortion law in Kenya allow abortion when the life or health of a woman is in danger [14]. Still, unsafe abortion remains the leading cause of maternal deaths in adolescent girls [13]. The contraceptive prevalence rate has increased steadily to 61.6 among married women aged 15–49 years in 2016 [15]. However, only 37% of sexually active adolescent girls aged 15–19 years are using a modern contraceptive method [12]. Consequently, the adolescent fertility rate is relatively high at 82 live births per 1000 women in comparison with the global fertility rate of 44.6 per 1000 women aged 15–19 [16].

Comprehensive sexuality education (CSE) is a key method for helping adolescents to achieve sexual health and rights and avoid negative health outcomes by providing accurate, realistic and non-judgmental information about sexuality and relationships [17]. There is clear evidence that CSE has a positive impact on adolescent sexual and reproductive health and prevents unintended adolescent pregnancies [18]. The Kenyan government supports CSE, yet education sector policies largely promote an abstinence-only approach. Teachers lack time, resources and knowledge on the topics, and students are taught that sex is dangerous and immoral for young people [19].

Abortion stigma has been researched among women with unintended pregnancies, as well as among health care providers [11, 20]. However, less is known about secondary school teachers’ and students’ views on adolescent pregnancy, abortion and contraceptive use. A deeper understanding of the constituents of stigma related to abortion and contraceptive use can help to design effective interventions to reduce such stigma and improve adolescents’ reproductive health. Research on teachers’ perspectives can facilitate implementation of CSE in secondary schools in this context. The aim of this study was to explore stigmatising attitudes related to abortion and contraceptive use among secondary school teachers and student peer-counsellors in a low-resource setting in western Kenya.

## Methods

### Study design

This mixed methods study was a prestudy nested in a cluster randomised intervention study, registered at ClinicalTrials.gov (NCT03065842), which aims to reduce stigma related to abortion and contraceptive use among secondary school students in Kisumu, Kenya. This study is based on a mixed methods approach in a convergent design according to Creswell et al. [21]. Qualitative and quantitative data were collected concurrently but separate, however analysed together, to inform one another on consistency and inconsistency. A triangulation procedure was used so that the quantitative insights could be validated by the qualitative data [22], and to reduce the risk of peer-pressure when discussing the topics in the FGDs. Four focus group discussions (FGDs) were conducted to capture teachers’ and student peer-counsellors’ attitudes towards abortion and contraceptive use among adolescent girls. Before commencing the FGDs, the respondents filled in two Likert scale questionnaires covering abortion and contraceptive use stigma to complement the qualitative findings. The reporting was done in accordance with the consolidated criteria for reporting qualitative research (COREQ) checklist [23].

### Study setting

The study was conducted in a low-income area of Kisumu East sub-county in Kisumu, western Kenya. Kisumu is the third biggest city in Kenya, with an estimated population of 500,000. One quarter (25%) of the total population in Kisumu County are between 10 and 19 years old. Kisumu County has a high proportion of primary school enrolment (95%), however, only 61% transition to secondary school. About 15% of adolescent girls aged 15–19 years have begun childbearing, which is slightly lower than the national level at 18% [24]. Most of the Kenyan population are Christian (90%) and a smaller proportion are Muslim (7%) [12].

One local secondary school was selected for the study based on a cluster-randomised procedure. The inclusion criteria were public secondary day schools with a minimum of 400 students (mixed gender) located in suburban areas within the study setting. Four schools were eligible for inclusion in the main intervention study. One intervention school and one control school were randomly drawn from a sample of these four schools. This study was conducted during the baseline data collection at the intervention school. The studied school had approximately 650 students and were run with Christian values.

The sexuality education at the study school, similar to most Kenyan secondary schools, is mainly taught in the subject of “life skills”. This is a non-examinable topic, hence there is little incentive for students and teachers to prioritise it. Most emphasis in the curriculum is placed on reproductive physiology and STI/HIV prevention [19]. The teaching focuses on abstinence, rather than discussing safe sex and practical skills related to contraceptive use. Premarital sex and abortion are presented as immoral activities. Little emphasis is put on equity and gender rights [19].

### Study respondents

Teachers and student peer-counsellors at the study school were invited to participate in the study. At the time of data collection, 35 teachers and 22 student peer-counsellors worked and studied at the school. Inclusion criteria for the teachers were to have worked at the school for at least 6 months prior to the start of data collection to ensure the teachers’ familiarity with the context of this particular school. Participation was voluntary and no specific teaching subject was required since the study focused on individuals’ views rather than their knowledge about the studied topics. Student peer-counsellors are selected by their classmates to be trustworthy representatives of the class and were therefore also invited to participate in the study on a voluntary basis. Inclusion criteria for the students were to be a student peer-counsellor for one of the classes at the study school. The respondents were invited to participate by a letter delivered to them by a research assistant.

Out of the 35 teachers working at the study school at the time of data collection, 29 met the inclusion criteria and 15 were available on the day of the FGDs, all of whom agreed to participate. Of the 22 student peer-counsellors, 21 were available on the day of the FGDs and they all agreed to participate.

### Data collection

The FGDs were conducted in private settings in classrooms at the study school in January 2017. The respondents were divided into four separate focus groups: female teachers (*n* = 8), male teachers (*n* = 7), female student peer-counsellors (*n* = 11) and male student peer-counsellors (*n* = 10). Four fieldworkers (two females and two males) in the research project acted as moderators and notetakers for the FGDs. Prior to the start of the data collection, the moderators and notetakers underwent a three-day theoretical and practical training in qualitative research methods with a focus on abortion and contraceptive use stigma, run by one female senior professor and one female PhD researcher (MM) representing Karolinska Institutet (KI), Sweden. The fieldworkers were postgraduates and undergraduates from Kisumu, who all had previous experience of working with sexual and reproductive health issues and of leading FGDs. The two female fieldworkers lead the two FGDs with female teachers and student peer-counsellors, while the two male fieldworkers lead the two FGDs with male teachers and student peer-counsellors. The first and second authors were active as note takers, while the last author overviewed the FGDs. None had any previous relationship with the respondents. The background and purpose of the research project were explained to the respondents before starting the FGDs.

Prior to commencing the FGDs, the respondents filled in two questionnaires to quantify their attitudes and beliefs about abortion and contraceptive use; the Stigmatising Attitudes, Beliefs and Actions (SABA) scale and the Contraceptive Use Stigma (CUS) scale. SABA is an 18-item scale developed in 2013 by Ipas and a validated tool to measure abortion stigma [25]. Three dimensions of abortion stigma are covered as subscales in the SABA scale: 1) negative stereotypes (8 items), 2) discrimination and exclusion (7 items) and 3) fear of contagion (3 items). Based on previous research within this project, the SABA scale was modified to address the perspective of adolescent girls [26]. This was done by replacing the word *woman* with *girl* in all items of the original scale. Furthermore, two items were added: *“A married woman is more deserving of an abortion than an unmarried woman”* and *“A girl who has had an abortion should be prohibited from going to school”* [26]. The modified SABA scale used in this study included a total of 20 items. Based on a similar framework, the Contraceptive Use Stigma (CUS) 7-item scale was developed within this project to measure stigmatising attitudes towards adolescent girls associated with contraceptive use [26]. The responses to the modified SABA and the CUS scales were set up on a Likert scale ranging from 1 *(‘strongly disagree’)* to 5 *(‘strongly agree’)*. Thus, the summarised score of the modified SABA scale ranged from a minimum of 20 to a maximum of 100, while the CUS scale ranged from a minimum of 7 to a maximum of 35. A higher score indicated more agreement with the statement and consequently higher stigma related to abortion and contraceptive use.

A topic guide for the FGDs to explore the respondents’ views on abortion and contraceptive use among adolescents was developed during two workshops in November 2016 and January 2017 (see Additional file 1). The guide was compiled based on the stigma framework of Shellenberg et al. [25]. The workshops were run in a collaboration between KI and Kisumu Medical Education Trust (KMET), a non-religious non-governmental organisation (NGO) based in Kisumu. Participants of the workshops were representatives from KI, KMET, and other local NGOs, as well as secondary school teachers and students, health care providers, and authorities from the Ministry of Education and the Ministry of Health in Kisumu County. During the second workshop, the topic guide was pilot tested on secondary school teachers and students who did not work or study at the study school.

The FGDs were performed in the afternoon after the last lecture of the school day to avoid interrupting ordinary lectures. The primary teaching language in Kenyan secondary schools is English, however, the moderators gave the respondents the option to also communicate in Kiswahili or in the local language Luo. Each discussion lasted around 65–95 min. A digital voice recorder was used to record the discussions, which were transcribed verbatim within 2 days thereafter. Only a few non-English words and phrases in Kiswahili or Luo came up in the FGDs, and they were discussed within the local research team to agree on the most accurate translation and then translated into English.

### Data analysis

The responses to the modified SABA and the CUS scales were categorised into 1–2 *(‘disagree’)*, 3 *(‘unsure’)* and 4–5 *(‘agree’)*. Descriptive statistics were used to summarise the total stigma scores. Mean and median values were calculated within each group to enable comparison of the groups. All statistical analyses were performed using SPSS 22.0.

The transcripts from the FGDs were analysed with Abductive Thematic Network Analysis (ATNA) using the ATLAS-ti software version 8. Abductive analyses require a two-step analysis [27]. First, the second author (SS) systematically examined the transcripts and identified codes. From the coded text segments, basic themes were identified and refined, according to Attride-Stirling [28]. Then, thematic networks were constructed by stratifying the themes into three different levels: basic themes, organising themes and global theme(s). Basic themes group codes of the same topics that are explicitly visible in the text. Organising themes represent clusters of basic themes centred on larger, shared issues. Finally, the global theme is the core, principal meaning of the whole content [28]. The second step involved re-examining the transcripts to evaluate the global theme to the raw data, to possibly identify parts of information that were not represented by the selected theme [27]. The first author (MH) scrutinised all transcripts and revised the analysis done by the second author. The themes were refined through a process of reflection and discussion between all authors and the local research team. A final global theme emerged that represented the main topics of the transcripts and framed the main discoveries. Throughout the analysis, all findings were discussed with the last author (MM), to improve the consistency and accuracy of the coding and the interpretation. The results were validated by two respondents of the FGDs.

## Results

A total of 36 teachers and student peer-counsellors participated in the study. The male teachers (*n* = 7) were aged 24–70 years, and the female teachers (*n* = 8) declined to share their ages. The male student peer-counsellors (*n* = 10) were aged 16–20 years, and the female student peer-counsellors (*n* = 11) were aged 15–18 years.

### The questionnaires

The total scores of the modified SABA and the CUS scales are summarised for each subgroup and the total study population in Table 1.

**Table 1:** Total scores of the modified SABA and the CUS scales shown for subgroups of teachers (*n* = 15), student peer-counsellors (*n* = 21) and the total

	Student female ***n*** = 11	Student male ***n*** = 8*	Teacher female ***n*** = 8	Teacher male ***n*** = 7	Total ***n*** = 34
**The modified SABA scale (min 20, max 100)**					
Range	35–61	25–47	23–51	30–62	23–62
Mean	43.7	37.3	36.9	42	40.2
Median	42	37	35.5	38	38
**The CUS scale (min 7, max 35)**					
Range	9–27	8–25	8–27	8–31	8–31
Mean	16.4	15.3	16.8	17.4	16.4
Median	14	15	16.5	15	15

*Two male students did not answer all questions in the questionnaires

*CUS* Contraceptive Use Stigma; *SABA* Stigmatising Attitudes, Beliefs and Actions

The complete results of the modified SABA scale (Table 2) show that 28 respondents out of 36 considered abortion a sin. Most of the female respondents (14/19) and half of the male respondents (9/17) thought that a girl who has an abortion brings shame to her family. Almost all (17/19 female, 15/17 male) respondents disagreed with that a married woman is more deserving of an abortion than an unmarried woman, however, 10/19 female and 8/17 male respondents believed that a girl who has had an abortion might be a bad influence on other girls. In general, most respondents disagreed with the items considering exclusion of girls who have had an abortion, e.g. 35 out of 36 respondents did not think that a girl who has had an abortion should be prohibited from going to school. In the CUS scale (Table 2), 6/19 female and 7/16 male respondents stated that a girl cannot decide for herself if to use a contraceptive method, and 5/19 female and 5/16 male respondents believed that a girl who uses contraception will encourage others to a promiscuous lifestyle. Regarding the risk of infertility, 6/19 female and 5/15 male respondents thought that a girl who uses contraceptives will have problems when she decides to get pregnant.

**Table 2:** The modified SABA and the CUS scales. Stigmatising attitudes towards adolescent girls associated with abortion and contraceptive use among teachers (n = 15) and student peer-counsellors (n = 21). The answers are shown for the total and separated by sex

Items: The modified SABA scale		**Disagree** Total* **(Female/Male)**	**Unsure** Total* **(Female/Male)**	**Agree** Total* **(Female/Male)**
1	A girl who has an abortion is committing a sin.	7 (3/4)	1 (1/0)	28 (15/13)
2	Once a girl has one abortion, she will make it a habit.	20 (10/10)	4 (2/2)	12 (7/5)
3	A married woman is more deserving of an abortion than an unmarried woman.	32 (17/15)	1 (1/0)	3 (1/2)
4	A girl who has an abortion cannot be trusted.	25 (13/12)	4 (3/1)	7 (3/4)
5	A girl who has an abortion brings shame to her family.	12 (4/8)	1 (1/0)	23 (14/9)
6	The health of a girl who has an abortion is never as good as it was before the abortion.	7 (2/5)	4 (3/1)	24 (14/10)
7	A girl who has an abortion is a bad mother.	26 (15/11)	6 (2/4)	4 (2/2)
8	A girl who has an abortion brings shame to her community.	15 (8/7)	2 (1/1)	19 (10/9)
9	A girl who has had an abortion should be prohibited from going to religious services.	34 (18/16)	2 (1/1)	0
10	A girl who has had an abortion should be prohibited from going to school.	35 (19/16)	1 (0/1)	0
11	A girl who has had an abortion should be teased so that she will be ashamed of her decision.	30 (16/14)	3 (1/2)	3 (2/1)
12	A girl should be disgraced in my community if she has had an abortion.	33 (18/15)	3 (1/2)	0
13	A man should not marry a woman who has had an abortion.	32 (18/14)	1 (1/0)	2 (0/2)
14	A girl who has had an abortion you should not be associated with.	36 (19/17)	0	0
15	A girl who has had an abortion should be pointed fingers at to let others know what she did.	35 (19/16)	0	1 (0/1)
16	A girl who has an abortion should not be treated the same as everyone else.	32 (17/15)	0	4 (2/2)
17	A girl who has an abortion can make other people fall ill or get sick.	34 (18/16)	2 (1/1)	0
18	A girl who has had an abortion might be a bad influence on other girls.	16 (9/7)	2 (0/2)	18 (10/8)
19	A girl who has an abortion should be isolated from other people in the community for at least 4 weeks after having an abortion.	33 (17/16)	3 (2/1)	0
20	If a boy has sex with a girl who has had an abortion, he will become infected with a disease.	31 (17/14)	5 (2/3)	0
Items: The CUS scale		**Disagree** Total* **(Female/Male)**	**Unsure** Total* **(Female/Male)**	**Agree** Total* **(Female/Male)**
21	A girl who uses a contraceptive method is promiscuous (sexually immoral, likes to have many sexual partners).	22 (12/10)	6 (2/4)	7 (5/2)
22	A girl who uses a contraceptive method will encourage to a promiscuous lifestyle.	21 (12/9)	4 (2/2)	10 (5/5)
23	A girl cannot decide for herself if to use a contraceptive method.	20 (12/8)	2 (1/1)	13 (6/7)
24	A married woman is more deserving of a contraceptive method than an unmarried woman.	18 (11/7)	4 (3/1)	13 (5/8)
25	A girl who uses contraceptives will have problems when she decides to get pregnant.	14 (7/7)	9 (6/3)	11 (6/5)
26	A girl who carries condoms is likely to have many sexual partners.	26 (12/14)	4 (3/1)	5 (4/1)
27	A girl should not insist to use a condom, it is the man to decide whether to use a condom or not.	33 (18/15)	1 (1/0)	1 (0/1)

*The internal drop-out rate for each item varied between 0 and 2

*CUS* Contraceptive Use Stigma; *SABA* Stigmatising Attitudes, Beliefs and Actions

### The FGDs

**Fig. 1:**
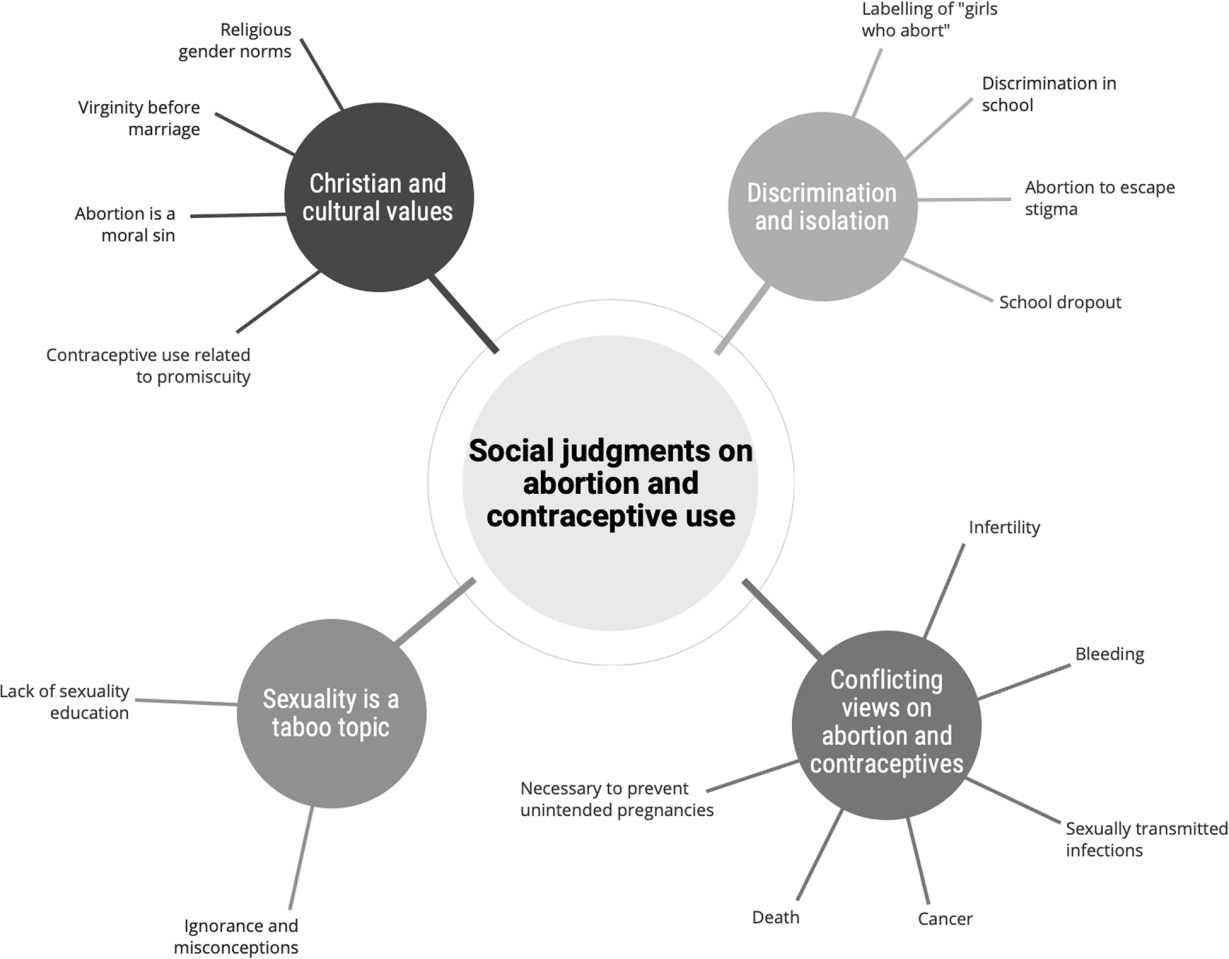
Overview of the themes identified in the qualitative thematic network analysis of four FGDs with teachers (*n* = 15) and student peer-counsellors (*n* = 21). The global theme: *Social judgments on abortion and contraceptive use* were merged from four organisational themes based on several basic themes as shown in the figure

An overview of the themes identified in the qualitative analysis is presented in Fig. 1. The initial analyses revealed several basic themes related to stigmatising attitudes towards young girls associated with abortion and contraceptive use. In deeper analysis the following four organisational themes were formed: Christian and cultural values, discrimination and isolation, conflicting views on abortion and contraceptives, and sexuality is a taboo topic. The organisational themes were merged into one global theme: *social judgments on abortion and contraceptive use*. Quotations from the FGDs are labelled ‘female or male, teacher or student, respondent number’, thus, ‘FT2’ is female teacher number 2, and ‘MS3’ is male student number 3.

### Christian and cultural values

Sexual intercourse before marriage was described as an unacceptable sin by the respondents, and a distinct separation was made between girls who had sex before marriage and girls who waited. The respondents explained that sexual intercourse before marriage is outside of the norm for a good Christian woman or wife, and sexual intercourse was considered a reproductive act for conceiving a child inside the nuclear family.

*In the African society and in Christianity, sex is very sacred. In most cases, sex is meant for procreation, but when these people are exposed to contraceptives, they will abuse sex, so sex loses its meaning. (FT2).*

Many respondents associated “girls who have sex before marriage” with negative characteristics and they were often described as promiscuous, irresponsible and unreliable. Accordingly, having an abortion or using contraceptives before marriage were seen as a sign of immoral behaviour.

*There is that shame associated with giving birth before marriage. It is due to stigmatization, they become social misfits in the society. They are being ridiculed, seen as irresponsible. (MT6).*

*I think when young people get used to using contraceptives, they develop a sense of immorality and they take sex for granted. And you know, it interferes with the morality. (FT4).*

*If you respect yourself, you will not engage in those sexual immoralities. (FS9).*

According to religious and cultural values in this community, virginity before marriage is expected. Both female and male respondents made clear that no good man will marry a woman who has had previous sexual intercourse. In a low-income setting, many parents’ main concern for their daughters is to get them married. A good marriage is favourable both for economic reasons as well as for the family’s reputation. The respondents mentioned several examples of when a girl might be mistreated by her parents if they would find out that she has been using contraceptives or had an abortion.

*When women reach the age of marriage and they have been using contraceptives, they will have broken their virginity. So, getting a serious man to marry you becomes a problem. (MS3).*

*Somebody could feel that “if I tell my mom, or if I tell my dad [about the pregnancy], they will think that I am not that trustworthy”, and because of that they go for abortion. (MT1).*

*There are some parents who don’t understand at all, they say that, “You are useless, worthless****,****I wonder if you will ever give birth again.” They treat them harshly because they know that this girl aborted without their consent. (FS8).*

Immoral behaviour during the adolescent years was by some respondents considered unforgivable and it was thought that the behaviour would become a part of the girls’ personality.

*When they start using contraceptives this early, what will they do when they get married? If they are using contraceptives it causes them to have multiple partners. When they get married, they don’t settle because they are already used to it. To them, it is not a big deal to have many men. And it gives them problem in marriage. Most of them don’t settle and they end up in divorce, because which man would allow a girl to go around like that. (FT8).*

*As my colleague said, they are viewed as promiscuous and loose; they are loose ladies. So, even after giving birth, even if the child is raised by the parents at the parental home, you realise that the community does not trust them. (MT5).*

### Discrimination and isolation

Just like girls who had sexual intercourse before marriage were separated from girls who waited, girls who had an abortion were also separated from other girls. In the FGDs, they were often labelled “girls who abort” by the respondents, a label commonly used for girls with a bad reputation in the community. However, some respondents described this as a common phenomenon even in the school environment, leading to discrimination and isolation of the labelled girls.

*In most cases, the other people in the society will look at a girl who has had an abortion differently. In some places they might judge her not only because they imagine this is a schoolgirl, they also imagine that this girl maybe had many partners or something like that. So, in most cases they are judged negatively. (FT3).*

*Some teachers like speaking about the students who have aborted, not knowing the reason for the abortion. They treat them like they should be isolated from the rest of us. (FS7).*

Some student respondents explained that discrimination could be explicit during lectures, and that some teachers have used the word “murder” to describe abortion.

*Some teachers have some bad habits, like if they know that a girl has aborted, they will just say something and when you pass, you hear, “this is the murderer”. (FS1).*

*Also, some teachers, if they know about your story, they always want to use you as an example to the others. In your presence or in your absence, they will always use you to refer to someone who did a certain thing that is bad, to show the other people that you did something wrong. (FS4).*

The social stigma surrounding an unintended pregnancy, especially for a young schoolgirl, might lead to abortion, according to the student respondents’ experiences. A secret abortion could be the only solution for a girl to escape this stigma.

*Some don’t want their status to be reduced, to be looked down upon. You see, some hold their heads high, so when they get pregnant, they don’t want me as a resident of that area to start laughing at them, so they have to do it [the abortion]. (MS5).*

Another consequence of unintended pregnancy among young girls was school dropout, which was an issue discussed by both teacher and student respondents. Situations where a young girl dropped out of school due to a pregnancy was said to happen every school year. Some teachers emphasised the need for a change and gave suggestions on possible ways to work with this issue.

*Actually, we realise that it [school dropout] is less among boys compared to the ladies. If someone was a bright student and they get pregnant, they drop out of school and their only hope was the education, then you realise that chance is wasted. (MT4).*

*I think a scenario should come whereby the pregnant teenagers that are, what we call, victims of teenage pregnancy, should be monitored so that they are not just left to go like that. They should be brought back to school and learn and then by the end of it all they become examples to the other fellows in the society. (MT7).*

### Conflicting views on abortion and contraceptives

The respondents considered abortion a dangerous procedure and thought that contraceptives might have negative effects on women’s health. Some respondents used these arguments to medically justify the social stigma surrounding abortion and contraceptive use. Mentioned consequences for abortion ranged from psychological trauma, bleeding and infertility to death.

*I think that they should not have an abortion, because it is harmful to their health. (FS11).*

*When a girl has an abortion, the girl tends to bleed a lot and she can become anaemic. (FS7).*

*The way I see it, when someone has an abortion, it may affect the uterus so that she will not become pregnant again and lose her fertility. (MS9).*

However, some respondents proposed situations when the risk of having an abortion could be worth taking.

*You see, abortion is not always a bad thing. Maybe the life of the mother is at stake; what if it’s either you abort the child, or you both die. (MS6).*

The most commonly mentioned risks with using contraceptives were acquiring sexually transmitted infections, infertility or cancer. Another believed negative effect of contraceptive use among young girls was that it might promote promiscuity.

*Contraceptives can interfere with the uterus, eventually damaging the uterus. Later in life when they want a child, they may not be able to get one. (FT1).*

*These many cancers that have come today, you find that some of them are related to those contraceptives. (FT7).*

*I’ve also heard that they come with internal reactions. You see, these contraceptives are chemical in nature. So, the body’s antibodies react differently. (MT2).*

*When girls are using contraceptives, maybe they will just expose themselves, “I am using them, so let me just have sex with any boy”. (FS10).*

The discussion on contraceptive use, similar to the one on abortion, brought up conflicting views within the focus groups. Some respondents expressed an acceptance towards contraceptive use among young unmarried women and argued on the importance of availability of contraceptives especially for school going students.

*“When it comes to contraceptives, initially my understanding was that contraceptives were supposed to be meant for the married people, maybe to control or to have family planning. But of late we have cases, even in our school, where young ladies are taken to some of these contraceptives by their parents, because the parents have tried to provide the guidance and counselling, all sorts of punishment, so that they can avoid this premature sexual behaviour. […]There is a lady in form two who gave birth after completing class eight. The mother is now struggling with both her daughter and a grandchild. So, you find that some parents opt to prevent further pregnancies by introducing these contraceptives to their young children. Maybe in my own opinion to some extent we need to accept contraceptives among the young ladies.” (MT4).*

### Sexuality is a taboo topic

Many teacher respondents expressed concerns about the lack of sexuality education provided to adolescent boys and girls. Sexuality, abortion and contraception are considered taboo topics and not even parents want to talk to their own children about it.

*In the African setup, mothers tend to leave the kids to learn sex education on their own. They don’t know that you can sit them down and tell them that “if you engage in this, this is what will happen”. (FT8).*

*We don’t talk openly about sexuality; mothers and fathers don’t want to talk openly about sexuality to their daughters – even issues related to condoms and sanitary towels. Especially not the lowly educated parents. (MT3).*

The lack of education on sexuality, abortion and contraceptive use leave a gap of ignorance among adolescents, something that both teacher and student respondents were aware of. To fill this knowledge gap, adolescent boys and girls seek information from other sources, often discussing the issues among their friends. The respondents gave several examples on how the lack of organised and accurate sexuality education increased the risk of misconceptions on abortion and contraceptive use.

*You find that if a person is not educated, instead of using contraceptives, she just does sex without using contraceptives. (FS2)*

*One thing I know is that they are students, so they still want to experiment with a lot of things that they hear. If they cannot control themselves, they must be enlightened about reproductive health. So, sexual reproduction education should be given to them, that is the first thing. Because they are ignorant about it. (MT1).*

To prevent this ignorance, several respondents wanted increased implementation of sexuality education in the school curriculum.

*In my opinion, girls and boys should be taught on the changes their bodies are undergoing, because some of them are unaware. So, they go out there and they don’t know what happens, and then they end up having unwanted pregnancies. (FT6).*

*I think it is high time that the ministry of education introduced sex education, so that our young girls and boys can understand what happens. Teaching on sexual education, and even on the use of condoms, should be allowed. (MT7).*

### Global theme: social judgments on abortion and contraceptive use

The analysis revealed the contextual aspects of stigma as a central topic in all organisational themes. The negative stereotypes and stigmatising attitudes towards girls associated with abortion and contraceptive use seem to be grounded in religious and cultural norms and beliefs in this community. In this context, femininity stands for chastity and purity before marriage, hence, any sign of breaking this norm, such as having an abortion or using contraceptives, put these girls in a category separate from other girls. These girls are quickly labelled as immoral, promiscuous, or as “girls who abort”. Integration of the results from the quantitative and qualitative data further emphasised these *social judgments on abortion and contraceptive use*, which may force adolescent girls to keep it secret and contribute to the high number of unsafe abortions.

*To Africans, an unwanted pregnancy is always shameful to the community. (MS2).*

## Discussion

This study explored stigmatising attitudes related to abortion and contraceptive use among secondary school teachers and student peer-counsellors in a low-resource setting in Kenya. Based on Christian and cultural values of femininity, the respondents considered abortion a sin and related contraceptive use to promiscuity. Discrimination and isolation of girls associated with abortion and contraceptive use was described as common in the community, as well as in the school setting, and school dropout was one consequence of unintended pregnancy. There were conflicting views on abortion and contraceptives, however many respondents considered both practices immoral and harmful, and medical arguments were used to justify these stigmatising attitudes. The respondents emphasised that sexuality is a taboo topic, which seem to cause ignorance and misconceptions on contraceptive use among adolescents. The respondents stressed the need of increased implementation of CSE in the school curriculum.

Worldwide, women who seek to terminate a pregnancy challenge the feminine ideals of female sexuality solely for procreation and compulsory motherhood, that originate in conservative gender roles and intend to control female sexuality [9]. The findings in this study show that premarital sex and abortion are considered sins in this context, thus adolescent girls associated with abortion and contraceptive use face social stigma and are often discriminated against. Similar findings have been shown previously in Kenya, as well as in many other contexts all around the world [11, 20, 29, 30]. Most respondents disagreed with the idea of discrimination against girls associated with abortion or contraceptive use, however expressed discriminating attitudes in the discussions. These girls were associated with negative attributes and often referred to as promiscuous and unreliable. Some student respondents claimed that teachers have used girls with unintended pregnancies or who have done an abortion as bad examples for the rest of the school. These girls were often labelled as “girls who abort”, implying that they will make abortion a habit. This belief was confirmed by the modified SABA scale where 16 of 36 respondents agreed or were unsure if a girl who has had one abortion will make it a habit.

Although some respondents were supportive of contraceptive use among adolescents, almost half of the respondents considered contraception to be mainly for married women. The results also revealed several misconceptions about contraceptives among both teacher and student respondents. Out of 34 respondents to this statement in the CUS scale, 20 respondents agreed or were unsure if a girl who uses contraceptives will have problems when she decides to get pregnant. This finding was confirmed in the FGDs, where many respondents stressed the risk of infertility after using contraceptives, especially in nulliparous women. This is a common misconception shown in previous studies [10, 31]. Other feared risks with using contraceptives were acquiring STIs or even cancer. These widespread misconceptions on contraception may be used as medical arguments to justify stigmatising attitudes and moral condemnation of premarital sexual relationships. This might be one of the explanations to the low use of modern contraceptives among sexually active adolescent girls in Kenya [12].

Furthermore, 14 of 35 respondents thought that a girl who uses contraception will encourage others to a promiscuous lifestyle. This came through in the FGDs in comments of the clear unacceptability of premarital sex, as well as an idea that sexual activity during the adolescent years would become a part of a person’s adult personality to continue having many sexual partners and be unfaithful in marriage. The results urge the need to increase quality sexual education in school to increase adolescents’ knowledge on sexual health and contraception, and openly discuss common views and ideas about sexuality in this context.

Most respondents believed that the health of a girl who has an abortion will never be as good as it was before the abortion. Infertility, bleeding and even fatal outcomes were mentioned as consequences of abortion in the FGDs. This might be explained by the high incidence of unsafe abortion, and consequently complications related to abortion, in Kenya [32]. Kenyan girls and women are aware of the risks of unsafe abortion, yet the social stigma surrounding unintended pregnancy may force them to choose a back-street abortion with unsafe procedures to keep it secret [33]. Respondents in this study confirmed that this is a choice that many young schoolgirls have to make and some showed understanding for situations when the risk might be worth taking, such as when the pregnant girl’s life is at risk or simply for the girl to be able to continue school.

In the modified SABA scale, a clear majority disagreed with the statement that a girl who has had an abortion should be prohibited from going to school. Although, in the FGDs, it was evident that female students associated with abortion and contraceptive use was looked down upon and discriminated against. The Kenyan government aims to implement CSE from primary school onwards [19], however, stigmatising attitudes among teachers might interfere with this. Previous research has shown that in school settings where time and resources are lacking, CSE is rarely prioritised [34]. Furthermore, teacher training is often limited in scope, and teachers feel uncomfortable to teach these sensitive topics [17]. To develop a sexual education programme for young people in a society that is deeply against premarital sex may be difficult, as it also may have negative consequences for the teacher, similar to the situation for health care providers who procure abortion. Societal pressure from colleagues and the society at whole may influence a teacher’s ability to provide sufficient CSE, even though the teacher themself might be supportive of sexuality education.

As shown in this study, where there is a knowledge gap, people tend to find explanations in common beliefs in the society. In this context, these ideas will many times be based on religious or cultural values. Hence, the current sexual education is often focused on abstinence, even though promoting abstinence is not proven to delay initiation of sexual intercourse or decrease unintended pregnancies [18]. One teacher respondent, quoted in the results, claimed that young people will always be curious and experiment whatever adults might tell them, and therefore promoted CSE. Many respondents, both teachers and students, were positive to increased implementation of CSE in Kenyan schools.

For future research, a similar study could be performed among a larger sample of secondary school teachers to further investigate their views. Since the findings in this study indicate that stigmatising attitudes towards adolescent girls related to abortion and contraceptive use are common among secondary school teachers in this setting, such study could provide a baseline for stigma reduction interventions among Kenyan teachers. Future studies could also include parents, and other members of the community such as church leaders, to explore their attitudes towards abortion and contraceptive use among young people.

### Limitations

This study was a prestudy nested in a larger study, as such it has a number of methodological weaknesses. However, the mixed methods approach allowed triangulation which facilitated validation and reliability (trustworthiness) of the data through cross-verification of the quantitative and qualitative data sources [35]. Nevertheless, the research topic was sensitive and we could not eliminate the risk of peer-pressure when discussing the topics in the FGDs. The trustworthiness was further hampered by the small sample size, due to limited time and financial resources in the prestudy phase. A larger sample size for the questionnaire-survey would allow a more rigorous analysis of the quantitative data, however, the main purpose of the questionnaires in this study was to explore consistency and inconsistency with the findings in the FGDs. Credibility, dependability and transferability are other core concepts for trustworthiness in qualitative research [36]. Credibility was gained by including both teachers and students of both sex and various ages to shed light on the discussed topics from different perspectives. To ensure that the respondents still would be as comfortable as possible to share their views on these sensitive topics, teachers and students were separated, and then further divided in groups based on sex. The student peer-counsellors might not represent all students’ views on the topics, however, were chosen as they represent each class of the school and have good insight in current discussions and issues in their class. How and when data saturation is reached is challenging to define in FGDs and could be analysed based on responses from individuals and the whole group [37]. We cannot assure we reached saturation, however we did find consistency between the two data sources. Transferability of the results to other settings are supported by consistency with other studies conducted in various contexts [4, 10, 11, 29, 34]. The most suitable themes to cover the data were discussed continuously within the research group during the analytic process, and representative quotations from the transcribed texts are provided to further strengthen the credibility of the results. Dependability was fortified by conducting the four FGDs simultaneously to avoid interactions between the participants.

## Conclusions

This study showed that adolescent girls associated with abortion and contraceptive use face social judgements and discrimination by secondary school teachers and fellow students in Kenya. Based on religious and cultural values of femininity, abortion was considered a sin, and girls using contraceptives were seen as promiscuous and immoral. Fear of social judgments on abortion and contraceptive use force adolescents to keep it secret and may contribute to the high number of unsafe abortions in Kenya. The results emphasise the need for continued implementation of CSE in Kenya. Further, sexual and reproductive health training needs to be implemented in teacher education to increase knowledge on adolescent sexuality, abortion and contraceptive use to improve adolescents’ sexual health and decrease the stigma.

## Supplementary information

**Additional file 1.**

## Data Availability

Additional data is available by emailing the last author marlene.makenzius@ki.se.

## Abbreviations

ATNA: Abductive thematic network analysis
COREQ: Consolidated criteria for reporting qualitative research
CSE: Comprehensive sexuality education
CUS: Contraceptive use stigma
FGD: Focus group discussion
KI: Karolinska Institutet
KMET: Kisumu Medical Education Trust
NGO: Non-governmental organisation
SABA: Stigmatising attitudes, beliefs and actions
