# Supplementary material for: Social judgments on abortion and contraceptive use: a mixed methods study among secondary school teachers and student peer-counsellors in western Kenya
Source: BMC Public Health. 2020 Apr 15;20:493. doi: 10.1186/s12889-020-08578-9 (PMC7161104; doi:10.1186/s12889-020-08578-9)
Supplement: Supplementary file 1 — Additional file 1. [file 12889_2020_8578_MOESM1_ESM.doc]

# FGD topics: Attitudes and Beliefs

Unintended pregnancy, Abortion and Contraception

1. **What do you think about unintended pregnancy among young girls in your community?**
2. **How do you think unintended pregnancy can be prevented?**

***Probes, if needed:*** Health care systems, community, **school**, church.

1. **What do you think are the reasons why some young girls and men don´t use contraceptives?**

***Probe:*** culture, religion, personal decisions.

1. **What do you think about benefits/risks connected to the use of contraceptives?**

***Probes:*** Short term, long term.

1. **Why do you think young girls have abortion?**

***Probes:*** When should it be allowed/not allowed (schools students, rape, married, etc)?

1. **How do you think a girl who have had an abortion are treated in the community?**

***Probes:*** Family, friends, health system, **schools**.

1. **In what way do you think an induced abortion can affect the health of the girl herself or other people in relation to her?**

***Probes:*** Physically, psychologically, economically, behaviour influences on others (**friends/peers**/family/partner).

1. **What role do male partners play in relation to contraceptive use?**

***Probes:*** Contraception decision, support.

1. **What role do male partner play in relation to induced abortion?**

***Probes:*** Abortion decision, support.

**Close the discussion:** We have now reached the end of this discussion. Is there anything else you think is important that we have not talked about today? Do you have any questions about what we talked about?

That concludes our focus group. Thank you so much for coming and sharing your thoughts and opinions with us.
